# The Perils of Practice

**Published:** 1886-02

**Authors:** Geo. L. Parmele

**Affiliations:** Hartford, Conn.


### THE PERILS OF PRACTICE.

PRESIDENT'S ADDRESS, DELIVERED BEFORE THE CONNECTICUT VALLEY DENTAL SOCIETY AT ITS TWENTY-SECOND ANNUAL MEETING, HELD IN SPRINGFIELD, MASS., NOV. 5 AND 6, 1885.

BY GEO. L. PARMELE, M. D., D. M. D., HARTFORD, CONN.

As an apt introduction and illustration of the ideas to which I wish to call your attention, let me repeat something read the other day:

"When a small boy, I was carrying a not very large ladder, when there was a crash. An unlucky movement had brought the rear end of my ladder against a window. Instead of scolding me, my father made me stop, and said very quietly: 'Look here, my son! there is one thing I wish you to remember, and it is that every ladder has two ends.' I never have forgotten it, though many years have gone by.

Do we not carry many things that have two ends besides ladders? Ah yes, every ladder has two ends, and it is a thing to be remembered in more ways than one."

Now gentlemen, even the ladder of dental practice has two ends, and one of them, that one to which many of you give too little heed, needs your careful attention. The end of the ladder where the rounds are composed of long, worrying, tedious hours, spent in close offices, making contortionists of yourselves in the earnest endeavor to ease and prevent the sufferings of others, and at the same time striving not to waste a moment in which you can earn reputation and an honest penny, you know all about; of that I need say nothing. But it is of that end of the ladder which points to a proper care of your own physical condition, both in the office and out in the pure air, that I wish you to think. That is the end to which, if neglected, a loud crash will sooner or later call your attention.

I do not wish to imply that you are not conscious of the other end of the ladder, neither is it my purpose to instruct as to the hygiene of professional life, with the laws of which you must be acquainted, but to remind the thoughtless that they cannot go on transgressing these laws without paying the penalty. No one will deny, I think, that maintenance of the body and mind in as nearly a normal condition as possible is worthy of more consideration than many of us give it.

The confinement in poorly ventilated rooms, breathing air poisoned by the exhalations of patients, many of them victims of disease, working for hours at a time in constrained positions, inflicting necessary pain on nervous patients, which, to a greater or less extent, causes nerve exhaustion in the operator, and the continuous concentration of vision upon minute objects, together with the constant worry and perplexity of business, are some of the reasons why those conscientiously engaged in dental practice will sooner or later break down, unless they take means to counteract these baneful influences.

One of the greatest evils of modern life is mental overwork, and there is no constitution so adamantine as to resist continuous confinement, weariness and worry. Work and play must alternate, for nature too severely tried is sure to call the balance and enforce a settlement. The adage "all work and no play makes Jack a dull boy" is one of those common sayings which we are bound to accept, whether we like it or not. Each violent emotion or train of thought operates upon the nutrition of the nerve centres, and continued worry and passion are more harmful than judicious hard labor.

With telegrams to keep them upon the *qui vive*, and express trains to hurry them hither and thither, Americans live at too high pressure. The influence of active competition is such that they think a holiday or outing cannot be afforded to relieve them of that intense, unremitting application, which leads to mental strain.

Members of our profession are no exception, and I frequently hear them say, "I cannot afford to lose the time." There is nothing they can better afford to do. The time spent in frequent outings, breathing "God's oxygen," is not lost, for owing to renewed vigor complete restitution is obtained for the seeming loss of time, by the larger amount and better quality of work accomplished in the remaining hours of labor. "I have sinned against my brother, the ass" (referring to his abused body), were the last words of St. Francis, of Assisi, when his self-inflicted martyrdom brought him to death's door.

We should all make a business of pleasure and recreation of mind and body. All medical men, who have given the subject any thought, agree as to the increase of nervousness in Americans, due to the chronic habit of constant excitement and push in our daily life, at home and abroad, sick or well. To hit the happy medium between under and overwork is no easy task. Many are injured by too little work. When what was once considered a cheerful task requires an extra effort for its accomplishment, and, as a direct outcome of a worried and flagging brain, errors and omissions commence to manifest themselves, it is a sad mistake to spur up the exhausted brain to increased vigor by artificial means. "That which one desires to do must not be his guide, but that which he has power to do."

So long as a brain worker can eat and sleep well, and takes abundant exercise in the open air, it is not necessary to impose special limits to his working hours. But when one begins to worry over business affairs, and about the numerous personal perplexities which we can seldom escape, his brain constantly contriving some way out of the difficulties which beset him admits of no quiet for sleep. Lack of the necessary rest soon disturbes the nervous system, and is quickly followed by dyspepsia and fits of depression, two of the principle miseries of an overworked body.

After all, it is worry that does the harm rather than work. I once heard the late Dr. Beard say that the three W's, Wine, Women and Worry, were the most fruitful causes of nervous shipwreck and insanity. I would not have you infer that I desire to picture dentistry as the most onerous of all labor, or that all who enter upon it are on a sure road to an early grave. Far from it, for I believe a properly conducted dental practice is one of the most pleasant of all special fields of labor. Still, there are circumstances connected with it which tend to render it particularly dangerous to those who go at it with too much push, and who neglect every hygienic law. The same amount of push would be much less injurious in many other occupations.

You all ought to know "what to do to be saved" better than I can tell you, and it seems unnecessary for me to lay down the laws of health. I only wish to spur up those who need it to a proper observance of those laws, and particularly to urge them to shorten their office hours, to take frequent holidays and outings, to devote themselves to some hobby which requires active exercise in the open air, and, in fact, for those inclined to overdo, I advise a cultivation of laziness as to business, and activity in all open air life.

The surest of all prophylactics is active exercise in the open air. Air is a part of our daily food, and by far the most important, the purity of our blood depending upon it. Jean Paul says : "On the day of judgment God will perhaps pardon you for starving your children when bread was dear, but if he should charge you with stinting them in his free air, what answer shall you make?"

Physical exercise, by accelerating the circulation of the blood, stimulates the activity of all those internal organs whose functions conjointly constitute the phenomena of life, and counteracts innumerable functional disorders, any one of which is sure to react on the nervous system.

Mirth is of value as a remedy. Men of a cheerful disposition are generally long lived, and anything tending to counteract the influence of worry and discontent contributes directly toward the preservation of health.

See to it that your offices and homes are in a proper sanitary condition, and let me suggest, where practicable, a reversal of the general rule, and that you make your operating rooms large and airy, and your reception rooms, if necessary, the smaller of the two.

Never eat until you have time to digest, for we are not nourished by what we eat, but by that which we assimilate.

Plenty of rest after meals is a good health rule. Wild animals, in obedience to instinct, seek out their hiding places after a heavy meal, and digest in peace. Let us follow their example. Digestion requires leisure. By attempting work while the stomach is full, we are not only unjust to ourselves, but to the labor we desire to perform.

In closing, I thank you for so patiently listening to this brief and unsatisfactory address. Let me also once more thank you for the compliment shown in bestowing upon me the highest honor in the power of this society. I feel deeply conscious of duties unfulfilled, for which I ask your forgiveness, but it is a pleasure to think that when I resign this chair it will be to one whom I feel sure will not disappoint your most sanguine expectations.

